# Supplementary material for: The schizophrenia associated protein DISC1 forms a multivalent tetrameric hub via conserved UVR dimers
Source: Nat Commun. 2026 Apr 17;17:5332. doi: 10.1038/s41467-026-71838-6 (PMC13273187; doi:10.1038/s41467-026-71838-6)
Supplement: Supplementary file 2 — Reporting Summary [file 41467_2026_71838_MOESM2_ESM.pdf]

Corresponding author(s): Jin Chuan Zhou

Last updated by author(s): Feb 14, 2026

Reporting Summary

Nature Portfolio wishes to improve the reproducibility of the work that we publish. This form provides structure for consistency and transparency in reporting. For further information on Nature Portfolio policies, see our Editorial Policies and the Editorial Policy Checklist.

Statistics

For all statistical analyses, confirm that the following items are present in the figure legend, table legend, main text, or Methods section.

n/a	Confirmed	
☐	☒	The exact sample size (*n*) for each experimental group/condition, given as a discrete number and unit of measurement
☐	☒	A statement on whether measurements were taken from distinct samples or whether the same sample was measured repeatedly
☒	☐	The statistical test(s) used AND whether they are one- or two-sided<br>*Only common tests should be described solely by name; describe more complex techniques in the Methods section.*
☒	☐	A description of all covariates tested
☒	☐	A description of any assumptions or corrections, such as tests of normality and adjustment for multiple comparisons
☐	☒	A full description of the statistical parameters including central tendency (e.g. means) or other basic estimates (e.g. regression coefficient) AND variation (e.g. standard deviation) or associated estimates of uncertainty (e.g. confidence intervals)
☒	☐	For null hypothesis testing, the test statistic (e.g. *F*, *t*, *r*) with confidence intervals, effect sizes, degrees of freedom and *P* value noted<br>*Give P values as exact values whenever suitable.*
☒	☐	For Bayesian analysis, information on the choice of priors and Markov chain Monte Carlo settings
☒	☐	For hierarchical and complex designs, identification of the appropriate level for tests and full reporting of outcomes
☒	☐	Estimates of effect sizes (e.g. Cohen's *d*, Pearson's *r*), indicating how they were calculated

Our web collection on statistics for biologists contains articles on many of the points above.

Software and code

Policy information about availability of computer code

Data collection	No custom code was used to collect the data in this study. The following commercially available software were used: EPU v2.5+ (Thermo Fisher Scientific) was used for both cryo-EM and negative stain EM data collection; AquireMP v2.5 (Refeyn) was used for mass photometry data collection.
Data analysis	No custom code was used to analyse the data in this study. The following open-source or commercially available software were used: cryoSPARC v4.7 (Structura Biotechnology) and DeepEMhancer v1.0 were used for cryo-EM data processing and analysis; Phenix v1.2, ISOLDE v1.8 (Altos Labs) and UCSF ChimeraX v1.8 were used for flexible fitting and refinement of the atomic model; MolProbity (as part of Phenix v1.2) was used for model validation; AlphaFold v3 was used for the structural prediction of DISC1 homologues. DiscoverMP v2024 R1 (Refeyn) was used for mass photometry data analysis; ASTRA v6 (Wyatt Technology) was used for SEC-MALS data analysis.

For manuscripts utilizing custom algorithms or software that are central to the research but not yet described in published literature, software must be made available to editors and reviewers. We strongly encourage code deposition in a community repository (e.g. GitHub). See the Nature Portfolio guidelines for submitting code & software for further information.

Data

Policy information about availability of data

All manuscripts must include a data availability statement. This statement should provide the following information, where applicable:

- Accession codes, unique identifiers, or web links for publicly available datasets
- A description of any restrictions on data availability
- For clinical datasets or third party data, please ensure that the statement adheres to our policy

The cryo-EM density maps generated in this study have been deposited in the Electron Microscopy Data Bank under the accession number EMD-54277 [https://www.ebi.ac.uk/pdbe/entry/emdb/EMD-54227] (DISC1core tetramer map). The atomic coordinates generated in this study have been deposited in the wwPDB database under accession code 9RUX [https://doi.org/10.2210/pdb9RUX/pdb] (DISC1core tetramer model). Raw mass photometry movies generated in this study have been deposited in the Molecular Biophysics Database under the record ID 1das6-9y357. Source Data are provided with this paper.

Research involving human participants, their data, or biological material

Policy information about studies with human participants or human data. See also policy information about sex, gender (identity/presentation), and sexual orientation and race, ethnicity and racism.

Reporting on sex and gender	This study did not involve human participants or human data.
Reporting on race, ethnicity, or other socially relevant groupings	This study did not involve human participants or human data.
Population characteristics	This study did not involve human participants or human data.
Recruitment	This study did not involve human participants or human data.
Ethics oversight	This study did not involve human participants or human data.

Note that full information on the approval of the study protocol must also be provided in the manuscript.

Field-specific reporting

Please select the one below that is the best fit for your research. If you are not sure, read the appropriate sections before making your selection.

☒ Life sciences ☐ Behavioural & social sciences ☐ Ecological, evolutionary & environmental sciences

For a reference copy of the document with all sections, see nature.com/documents/nr-reporting-summary-flat.pdf

Life sciences study design

All studies must disclose on these points even when the disclosure is negative.

Sample size	No statistical methods were used to predetermine sample size. The size of the data collected for the cryo-EM experiment was chosen such that an EM map of about 4A overall resolution could be derived. Such global resolution enabled confident model building for the majority of the map. The measurement concentration for mass photometry, which determines the number of protein molecules quantified by mass, was optimized to provide sufficient counts and accurate mass estimation in accordance with the standard protocol (Kratochvil J et al., Nature Protocols, 2025).
Data exclusions	No data were excluded in this study, except for where the removal of junk or sub-quality micrographs/particles is an essential part of the EM data processing pipeline.
Replication	Well-established statistical methods (e.g. maximum-likelihood optimisation) are inherent to the cryo-EM processing pipeline used in this study. They ensure that any noise is expected to contribute minimally to the overall result. Therefore, data reproducibility is generally robust. For each mass photometry sample, at least three separate measurements were taken, yielding closely similar values. For MALS, at least two independent measurements were performed, yielding very similar results. For in vitro reconstitution, at least two separate experiments were carried out and verified by negative staining, yielding similar results.
Randomization	Automated data randomization is inherent to several steps within the cryo-EM data processing pipeline used in this study. For example, as defined by the gold-standard FSC estimation of EM map resolution, two randomly selected half-sets were employed here. For assays such as mass photometry and MALS, a random population of the sample is measured at each time.
Blinding	Blinding is not necessary for cryo-EM data analysis, as knowledge of the target molecule by the experimenter does not affect the overall processing result which rely mostly on software algorithm-based calculations. Similarly, for the mass photometry experiment, knowledge of the sample identity has no impact on how the counting events are recorded.

Reporting for specific materials, systems and methods

We require information from authors about some types of materials, experimental systems and methods used in many studies. Here, indicate whether each material, system or method listed is relevant to your study. If you are not sure if a list item applies to your research, read the appropriate section before selecting a response.

Materials & experimental systems

n/a	Involved in the study
☒	☐ Antibodies
☒	☐ Eukaryotic cell lines
☒	☐ Palaeontology and archaeology
☒	☐ Animals and other organisms
☒	☐ Clinical data
☒	☐ Dual use research of concern
☒	☐ Plants

Methods

n/a	Involved in the study
☒	☐ ChIP-seq
☒	☐ Flow cytometry
☒	☐ MRI-based neuroimaging

Plants

Seed stocks	No plants or seeds were used for this study
Novel plant genotypes	No plants or seeds were used for this study
Authentication	No plants or seeds were used for this study
