# Supplementary material for: The schizophrenia associated protein DISC1 forms a multivalent tetrameric hub via conserved UVR dimers
Source: Nat Commun. 2026 Apr 17;17:5332. doi: 10.1038/s41467-026-71838-6 (PMC13273187; doi:10.1038/s41467-026-71838-6)
Supplement: Supplementary file 3 — Transparent Peer Review file [file 41467_2026_71838_MOESM3_ESM.pdf]

nature portfolio

Peer Review File

The schizophrenia associated protein DISC1 forms a multivalent tetrameric hub via conserved UVR dimers

Corresponding Author: Dr Jin Zhou

This file contains all reviewer reports in order by version, followed by all author rebuttals in order by version.

Version 0:

Reviewer comments:

Reviewer #1

(Remarks to the Author)
In this paper, the authors present the structure of the mouse DISC1 322-722 domain solved by cryo-electron microscopy. They identify a particular tetrameric “bow tie” structure and make predictions that are tested by mutations. Overall this is a highly interesting paper that is worth being published.

I have a few points that I believe would strengthen the paper by integrating previous work (which is neglected in the present version) and important questions that might direct the reader to future work; These concern mainly the fact that the authors investigated mouse DISC1 but disease-relevant seems to be the human DISC1 to which there is only about 65% amino acid homology in the named domain (all of this should be critically discussed):

Why did the authors choose to express mouse and not human DISC1 322-722? My hunch is that, as previous work indicated (PMID 28249940 and 34921141 should be discussed), the human pendant is insoluble? For example, a tetrameric structure in an overlapping domain was already discussed for human DISC1 in PMID 28249940 – this should be credited.

Given the apparent problems with insolubility that the authors also mention in the introduction, it would be worthwhile discussing how the soluble tetrameric structure could be overrun to become insoluble by events or interactions (PMID 16299498, 28249940 and 34921141).

In this context, it would be interesting if the authors commented on the primate specific exon 11 insertion (not part of the 322-722 domain, e. g. PMID 16965828) but relevant for linking structural effects to disease.

Reviewer #2

(Remarks to the Author)
Zhou and colleagues set out to determine the structure of the ordered region of DISC1, an important scaffolding protein that is associated with a host of mental illnesses. Using cryo-EM, the authors find that DISC1 forms a dimer of dimer structure with two fold symmetry and shares structural conservation with a primarily bacterial UVR domain. This manuscript is easy to read and the data presented is clear. I suggest this manuscript be accepted, pending a few points below.

1. My only real suggestion would be to validate the structure and the tetramerziation interface with mutagenesis, with a particular focus on the interaction between CC1 and CC3. This is a pretty straightforward structure paper, but the authors don't go the final step and validate the novel hydrophobic interface and salt bridges with point mutagenesis.

2. Another minor qualm I have is that the way that the discussion is written makes it seem that Zhou and colleagues are the first to suggest that DISC1 is a scaffold protein. However, DISC1 has been suggested to be a scaffold that facilitates the coordination of multiple pathways for a long time. For one example, see Yerabham et al., 2013. The manuscript should be rewritten to reflect that this model was already proposed by many others previously.

Reviewer #3

(Remarks to the Author)
Zhou and co-workers report the cryo-EM structure of the conserved core region of DISC1, a scaffold protein strongly associated with mental illnesses including schizophrenia and depression. The authors demonstrate that DISC1 constitutively assembles into a homotetramer. Interestingly, the structure reveals an overall two-fold, rather than four-fold, symmetry, with the four DISC1 copies arranged in a "dimer of dimers" conformation, where the two protomers within each dimer adopt distinct structures. As predicted from bioinformatics, the DISC1core architecture resembles UVR domains found in bacterial proteins. The authors further characterize interactions between DISC1 and its binding partner NDE1, which engages the C-terminal tail of the protein, and show that NDE1 binding is independent of tetramerization. This structural and biochemical analysis provides a framework for understanding how disease-associated mutations at the DISC1 locus perturb folding and partner interactions.

Overall, this is a focused and well-executed study that combines cryo-EM and biochemistry to advance our understanding of the molecular architecture of DISC1. The manuscript is clearly written and will be of interest to structural biologists and neuroscientists. A few revisions would improve clarity and impact.

Major points:
1. The authors suggest that the clinically relevant t(1;11) truncation event might affect the folding of the DISC1core and therefore DISC1 oligomerisation. This hypothesis could be directly tested by recombinantly expressing the equivalent murine construct and assessing its oligomerization state (e.g., by mass photometry).
2. The manuscript would benefit from a supplementary figure showing the model-to-density fit, particularly for the critical interactions described in Figures 2 and 3. The data quality appears excellent, and including this would allow readers to assess the evidence for the structural claims without relying solely on deposited maps.

Minor points:
1. Figure labels are often too light (e.g., cyan or yellow in Figs. 1c, 1e, 4d) or too small (e.g., MP plot labels in Fig. 5b-c). Improving readability would make the figures more accessible.
2. Supplementary fig. 2a: please provide here as well the total number of particles picked, and the number of 2D classes used for the 4x classification. The 2D classes used for template picking should be displayed. It would also be helpful to show examples of classes which were rejected.
3. Supplementary fig. 2a: Two different classes from heterogeneous refinement were used. Are there any differences between them?
4. Fig. 5a: overlaying the DISC1 cartoon on top of the object of interest complicates understanding and makes the inset functionally useless. A less intrusive overlay would be useful.
5. Figure 6: while visually appealing, this figure has limited connection to the presented data. PDE4B and TRAK1/2 are only mentioned here, and GSK3β is not discussed in the text. A more useful summary would showcase how DISC1 interactions and known genetic mutations map on the architecture of the DISC1 tetramer.

Reviewer #4

(Remarks to the Author)
I co-reviewed this manuscript with one of the reviewers who provided the listed reports. This is part of the Nature Communications initiative to facilitate training in peer review and to provide appropriate recognition for Early Career Researchers who co-review manuscripts.

Version 1:

Reviewer comments:

Reviewer #1

(Remarks to the Author)
All issues raised have been addressed sufficiently. The additional experiments proposed by other reviewers have furthermore contributed to improve the manuscript. Congratulations for this interesting work.

Reviewer #2

(Remarks to the Author)
The authors did a great job addressing my concerns. I have no further issues and think it is ready for publication.

Reviewer #3

(Remarks to the Author)
The authors have responded to our comments and we are happy for the paper to be published.

Reviewer #4

(Remarks to the Author)

I co-reviewed this manuscript with one of the reviewers who provided the listed reports. This is part of the Nature Communications initiative to facilitate training in peer review and to provide appropriate recognition for Early Career Researchers who co-review manuscripts.

Open Access This Peer Review File is licensed under a Creative Commons Attribution 4.0 International License, which permits use, sharing, adaptation, distribution and reproduction in any medium or format, as long as you give appropriate credit to the original author(s) and the source, provide a link to the Creative Commons license, and indicate if changes were made.
In cases where reviewers are anonymous, credit should be given to 'Anonymous Referee' and the source.
The images or other third party material in this Peer Review File are included in the article's Creative Commons license, unless indicated otherwise in a credit line to the material. If material is not included in the article's Creative Commons license and your intended use is not permitted by statutory regulation or exceeds the permitted use, you will need to obtain permission directly from the copyright holder.
To view a copy of this license, visit https://creativecommons.org/licenses/by/4.0/

Authors: We are extremely grateful to all reviewers for their insightful and constructive comments. Their helpful suggestions allowed us to further improve the manuscript by way of additional supporting data as well as by providing a more comprehensive discussion over the important implications of our findings.

REVIEWER COMMENTS

Reviewer #1 (Remarks to the Author):

In this paper, the authors present the structure of the mouse DISC1 322-722 domain solved by cryo-electron microscopy. They identify a particular tetrameric “bow tie” structure and make predictions that are tested by mutations. Overall this is a highly interesting paper that is worth being published.

Authors: We thank the reviewer for their careful evaluation and positive comments.

I have a few points that I believe would strengthen the paper by integrating previous work (which is neglected in the present version) and important questions that might direct the reader to future work; These concern mainly the fact that the authors investigated mouse DISC1 but disease-relevant seems to be the human DISC1 to which there is only about 65% amino acid homology in the named domain (all of this should be critically discussed):

Authors: We are very much grateful for the reviewer’s insightful comments and for raising the important topic of DISC1 evolutionary conservation (discussed by ref. 43 main text, PMID 16965828, among others). We take the opportunity to highlight that the ~65% sequence identity in $DISC1_{core}$ between human and mouse does not fully capture the much higher functional conservation existing between the two species. Indeed, when considering the side chain properties of the residues in this region, roughly 90% of the sequence show conserved hydropathy between human and mouse homologues, which in turn, we argue, translates into a conservation of structure. Indeed, negative stain EM analysis of recombinantly expressed human $DISC1_{core}$ showed 2D classes predominantly featuring the same bow-tie architecture characteristic of the murine DISC1 structure (Supp Fig. 6g). Nonetheless, the human DISC1 particles were not as well behaved as the mouse counterparts under our grid freezing conditions. Therefore, we were only able to derive a high resolution cryo-EM map for the mouse protein. We now show the EM data in Supp Fig. 6g and have included the relevant discussion under paragraph, line 379-385, in the Discussion.

Why did the authors choose to express mouse and not human DISC1 322-722? My hunch is that, as previous work indicated (PMID 28249940 and 34921141 should be discussed), the human pendant is insoluble? For example, a tetrameric structure in

an overlapping domain was already discussed for human DISC1 in PMID 28249940 – this should be credited.

Authors: We confirm that the human equivalent of mouse $DISC1_{core}$ can be expressed and purified in solution under the same conditions described in our Methods section. We note however that the presence of a small globular tag like GB1, as in the case of our mouse $DISC1_{core}$ construct for example, may aid in sample solubility. Indeed, we refrained from removing the tag during our structural studies as we reasoned it could be beneficial for maintaining protein stability prior to grid freezing.

We are grateful to the reviewer for highlighting the importance of acknowledging previous studies on DISC1 oligomerisation properties. We have now added proper citation of those early pioneering work (including ref. 32 main text, PMID 28249940) under paragraph, line 300-310. While previous biochemical and biophysical studies were remarkable in providing a systematic analysis of individual DISC1 domains, our study now presents a more comprehensive picture of the interactions among those domains from a structural standpoint. We believe our structural model mostly captures the molecular architecture of the largest and possibly one of the most prevalent *in vivo* isoforms, known as L (ref. 34 main text). However, it would be also interesting for future studies to investigate the molecular architecture of other DISC1 variants. It is highly possible that some of the previously identified oligomeric modes may become dominant in those cases.

Given the apparent problems with insolubility that the authors also mention in the introduction, it would be worthwhile discussing how the soluble tetrameric structure could be overrun to become insoluble by events or interactions (PMID 16299498, 28249940 and 34921141).

Authors: We thank the reviewer for raising an interesting point here in regard to conditions where DISC1 could become insoluble. In the Discussion session, we now specifically comment about the aggregation propensity of two clinically relevant mutant forms (the t(1;11) and the 4bp frameshift mutations, ref. 5 main text) under paragraph, line 342-354. An interesting common feature underlying the two mutants is the fact that both deletion events fall within a coiled coil domain (CC3 and DISC1-C respectively). For both domains, structural data predicts the exposure of normally buried hydrophobic residues that can potentially contribute to non-specific aggregates.

In this context, it would be interesting if the authors commented on the primate specific exon 11 insertion (not part of the 322-722 domain, e. g. PMID 16965828) but relevant for linking structural effects to disease.

Authors: The reviewer raises another interesting point regarding the link between splicing events and potential perturbations to DISC1 structure and consequently

complex stability. The specific splicing variant, Lv, which the reviewer refers to, lacks a short sequence located C-terminally to DISC1$_{core}$ and therefore beyond the boundaries of our current structure. In the absence of any structural information regarding this particular isoform, it is difficult to predict how the splicing event may alter DISC1 oligomeric state and/or biochemical stability. Although our work at present lacks the information to adequately address these aspects of DISC1 biology, we do believe the structure presented by this study offers a solid basis for the field to start exploring those questions. We make mention of this under paragraph, line 373-378.

Reviewer #2 (Remarks to the Author):

Zhou and colleagues set out to determine the structure of the ordered region of DISC1, an important scaffolding protein that is associated with a host of mental illnesses. Using cryo-EM, the authors find that DISC1 forms a dimer of dimer structure with two fold symmetry and shares structural conservation with a primarily bacterial UVR domain. This manuscript is easy to read and the data presented is clear. I suggest this manuscript be accepted, pending a few points below.

Authors: We thank the reviewer for their careful evaluation and positive comments.

1. My only real suggestion would be to validate the structure and the tetramerziation interface with mutagenesis, with a particular focus on the interaction between CC1 and CC3. This is a pretty straightforward structure paper, but the authors don't go the final step and validate the novel hydrophobic interface and salt bridges with point mutagenesis.

Authors: We thank the reviewer for raising the important point of validating the novel CC1$_{UVR}$ and CC3 $_{UVR}$ interface that we uncovered and structurally characterised. Following their suggestion, we have now purified a mutant, DISC1 ΔN$_{mut\ C}$ where four residues, namely V366, Y371, L593 and F598, found at the hydrophobic core of this interface, have been mutated. We show in Supp. Fig 5 that DISC1 ΔN$_{mut\ C}$ is deficient in tetramerization as only monomeric and dimeric species could be detected by mass photometry. This result is rather striking given the extent of contacting residues mediating this interface and further highlights the critical roles played by those four residues in maintaining an integral DISC1$_{core}$ assembly. Alongside with our DISC1 ΔN$_{mut\ A}$, which targets residues mediating dimer-dimer interaction, both major interfaces contributing to DISC1 overall tetramer fold have now been experimentally validated.

2. Another minor qualm I have is that the way that the discussion is written makes it seem that Zhou and colleagues are the first to suggest that DISC1 is a scaffold protein. However, DISC1 has been suggested to be a scaffold that facilitates the coordination of multiple pathways for a long time. For one example, see Yerabham et

al., 2013. The manuscript should be rewritten to reflect that this model was already proposed by many others previously.

Authors: It is not our intention to be misleading in our discussion and we fully acknowledge the need, as rightly highlighted by the reviewer, to properly reference studies where the model of DISC1 as a scaffold has been proposed before. In the Discussion section, we have now added a number of references (including Yerabham ASK et al, 2013, ref. 37 main text, line 312) where the scaffold model was previously reviewed. We limit our own discussion to the fact that our structure now provides a molecular framework that lends strong support to the scaffold model. In reflection of this, we have also rephrased the last sentence in the Introduction section (line 94-95). Lastly, we have removed some comments in relation to other binding partners which are not the focus of our current study.

Reviewer #3 (Remarks to the Author):

Zhou and co-workers report the cryo-EM structure of the conserved core region of DISC1, a scaffold protein strongly associated with mental illnesses including schizophrenia and depression. The authors demonstrate that DISC1 constitutively assembles into a homotetramer. Interestingly, the structure reveals an overall two-fold, rather than four-fold, symmetry, with the four DISC1 copies arranged in a “dimer of dimers” conformation, where the two protomers within each dimer adopt distinct structures. As predicted from bioinformatics, the DISC1core architecture resembles UVR domains found in bacterial proteins. The authors further characterize interactions between DISC1 and its binding partner NDE1, which engages the C-terminal tail of the protein, and show that NDE1 binding is independent of tetramerization. This structural and biochemical analysis provides a framework for understanding how disease-associated mutations at the DISC1 locus perturb folding and partner interactions.

Overall, this is a focused and well-executed study that combines cryo-EM and biochemistry to advance our understanding of the molecular architecture of DISC1. The manuscript is clearly written and will be of interest to structural biologists and neuroscientists. A few revisions would improve clarity and impact.

Authors: We thank the reviewer for their comprehensive summary and positive assessment of our study.

Major points:

1. The authors suggest that the clinically relevant t(1;11) truncation event might affect the folding of the DISC1core and therefore DISC1 oligomerisation. This hypothesis could be directly tested by recombinantly expressing the equivalent murine construct and assessing its oligomerization state (e.g., by mass photometry).

Authors: We thank the reviewer for raising a very important point in terms of the relevance of our structural data in relation to the notorious t(1;11) disease mutation that led to early research interest in the DISC1 protein. We have now purified a mouse construct, DISC1 $\Delta N_{mut\ B}$ (aa. 322-595), that recapitulates the truncation effect suffered by the human t(1;11) mutant. In agreement with our structure-based prediction, DISC1 $\Delta N_{mut\ B}$ exists as both monomer and dimers in solution but is deficient in tetramer assembly (Supp Fig. 5). Notably, this particular mutant required the addition of a MBP solubility tag in order for it to be purified in solution hinting to the aggregation propensity introduced by the mutation. This is to be expected since our structure predicts exposure of normally buried hydrophobic residues in the CC3 domain as a consequence of the truncation event.

2. The manuscript would benefit from a supplementary figure showing the model-to-density fit, particularly for the critical interactions described in Figures 2 and 3. The data quality appears excellent, and including this would allow readers to assess the evidence for the structural claims without relying solely on deposited maps.

Authors: We thank the reviewer for carefully assessing our deposited atomic model and EM map. To facilitate readers assessment of the correlation between our model and the EM data, we have now added Supp Fig. 3 where the model-to-map fit for both major DISC1 tetramerisation interfaces are shown in detail.

Minor points:
1. Figure labels are often too light (e.g., cyan or yellow in Figs. 1c, 1e, 4d) or too small (e.g., MP plot labels in Fig. 5b-c). Improving readability would make the figures more accessible.

Authors: To enhance readability, we have now systematically increased the colour intensity as well as font size and boldness of the labels used across all figures including the ones highlighted by the reviewer.

2. Supplementary fig. 2a: please provide here as well the total number of particles picked, and the number of 2D classes used for the 4x classification. The 2D classes used for template picking should be displayed. It would also be helpful to show examples of classes which were rejected.

Authors: We have now edited Supp Fig. 2a to include:

- The total number of particles picked;
- The number of 2D classes set for each round of classification;
- Examples of 2D templates that were used as references for the template picking job;
- Examples of 2D classes whose particles were excluded from the 3D reconstruction part of data processing.

3. Supplementary fig. 2a: Two different classes from heterogeneous refinement were used. Are there any differences between them?

Authors: We would like to clarify that both 3D class 1 and 4 highlighted in Supp Fig. 2a represent the same DISC1 conformation. Indeed, the number of particles contributing to each of those two classes are roughly similar (114,821 particles in class 1 compared to 112,319 particles in class 4). For this reason, the two particle stacks were pooled together prior to downstream processing.

4. Fig. 5a: overlaying the DISC1 cartoon on top of the object of interest complicates understanding and makes the inset functionally useless. A less intrusive overlay would be useful.

Authors: We have now edited Fig 5a such that the cartoon representation is next to the inset and only a dashed line tracing the boundaries of the DISC1 tetramer is now overlaying the micrograph inset.

5. Figure 6: while visually appealing, this figure has limited connection to the presented data. PDE4B and TRAK1/2 are only mentioned here, and GSK3β is not discussed in the text. A more useful summary would showcase how DISC1 interactions and known genetic mutations map on the architecture of the DISC1 tetramer.

Authors: We acknowledge that the reviewer makes a fair point here about the relevance of our original summary figure with respect to the key findings presented by the study. We have now completely overhauled the summary figure to focus instead on how our $DISC1_{core}$ structure may be interpreted in the context of both the t(1;11) disease mutant and the more common SNP variants associated with schizoaffective disorders. In contrast to the previous version, we now only highlight the NDE1 binding site in the figure as this best reflects the focus of the current study. As a result of the changes in Fig. 6, we have also modified the Discussion section to provide more analysis on the impact of disease mutations and variants on DISC1 structure (paragraphs, line 328-372).

Reviewer #4 (Remarks to the Author):

I co-reviewed this manuscript with one of the reviewers who provided the listed reports. This is part of the Nature Communications initiative to facilitate training in peer review and to provide appropriate recognition for Early Career Researchers who co-review manuscripts.

Authors: We express our gratitude to the reviewer for contributing to the critical evaluation of our manuscript.

Authors: We thank all the reviewers for their very supportive and positive comments.

REVIEWER COMMENTS

Reviewer #1 (Remarks to the Author):

All issues raised have been addressed sufficiently. The additional experiments proposed by other reviewers have furthermore contributed to improve the manuscript. Congratulations for this interesting work.

Reviewer #2 (Remarks to the Author):

The authors did a great job addressing my concerns. I have no further issues and think it is ready for publication.

Reviewer #3 (Remarks to the Author):

The authors have responded to our comments and we are happy for the paper to be published.

Reviewer #4 (Remarks to the Author):

I co-reviewed this manuscript with one of the reviewers who provided the listed reports. This is part of the Nature Communications initiative to facilitate training in peer review and to provide appropriate recognition for Early Career Researchers who co-review manuscripts.
